# “To implant or not to implant”: electrically evoked auditory brainstem response audiometry for decision-making in vestibular schwannoma resection with CI

**DOI:** 10.1007/s00106-024-01471-6

**Published:** 2024-04-22

**Authors:** Valerie Dahm, Anselm Joseph Gadenstaetter, Christoph Arnoldner

**Affiliations:** Department of Otorhinolaryngology, Head and Neck Surgery, Vienna General Hospital, Medical University of Vienna, Waehringer Guertel 18–20, 1090 Vienna, Austria

**Keywords:** Neoplasm, Cochlear implantation, Hearing loss, Electrophysiology, Monitoring, Neoplasie, Cochleaimplantation, Hörverlust, Elektrophysiologie, Monitoring

## Abstract

Vestibular schwannomas (VS) are often associated with debilitating hearing loss. Therefore, preservation and rehabilitation of hearing have become major therapeutic goals of VS management. Recently, cochlear implantation (CI) has been established as an effective treatment option for VS-associated hearing loss. Nevertheless, the integrity and proper function of the cochlear nerve must be evaluated before conducting CI to ensure optimal CI outcomes. Various methods to determine cochlear nerve integrity and functionality have emerged in the last few years. Of these, the use of electrically evoked auditory brainstem response audiometry (eABR) in particular has been proven to be a meaningful tool for monitoring cochlear nerve health during VS surgery. Here, the cochlear nerve can be electrically stimulated using an intracochlear test electrode before, during, and after tumor extirpation. Subsequently, the resulting brainstem responses can be measured and interpreted accordingly to obtain direct information on the cochlear nerve function. This allows for continuous monitoring of cochlear nerve function throughout the course of VS surgery and aids in the decision-making for CI candidacy. Finally, in the case of preserved brainstem responses, CI can be performed instantly after VS extirpation. This simultaneous approach offers several advantages over two-staged procedures and has been shown to be an efficient and safe procedure for restoring hearing after VS removal.

## Abstract (de)

Hörverlust stellt eines der schwerwiegendsten Symptome von Vestibularisschwannomen (VS) dar, das mit sehr hoher Prävalenz einhergeht. Da die überwiegende Anzahl der Patient:innen mit VS an Hörverlust leiden, sind der Hörerhalt und Rehabilitation des Hörvermögens zu zentralen Aufgaben der VS-Therapie geworden. Vor allem in den letzten Jahren hat sich hier die Cochleaimplantation (CI) als besonders effektive Maßnahme zur Wiederherstellung des Hörvermögens etabliert. Vor jeder CI sollte jedoch die Integrität des Hörnervs überprüft werden, um einen Therapieerfolg gewährleisten zu können. Um die Funktionsfähigkeit des Hörnervs zu überprüfen, stehen verschiedenste Methoden mit unterschiedlichster Aussagekraft zur Verfügung. Insbesondere die Messung elektrisch evozierter auditorischer Hirnstammpotenziale (eBERA) im Rahmen der VS-Operation hat sich hier als erfolgreiche Methode bewährt, um die Funktion des Hörnervs bestimmen zu können. Hierbei wird der Hörnerv mithilfe einer intracochleär platzierten Testelektrode elektrisch stimuliert, wodurch daraus resultierende Hirnstammpotenziale abgeleitet werden können. Dies kann sowohl vor, während als auch nach der Tumorexstirpation erfolgen und erlaubt somit eine kontinuierliche Überwachung der Funktion des Hörnervs. Bei erhaltenen Potenzialen nach Tumorentfernung kann schlussendlich auch direkt intraoperativ anschließend an die VS-Exstirpation eine CI durchgeführt werden. Dieses simultane Vorgehen hat sich besonders zur Vermeidung von Zweitoperationen und der hiermit assoziierten Risiken bewährt und stellt eine effektive Möglichkeit dar, das Hörvermögen von Patient:innen mit VS wiederherzustellen.

Vestibular schwannomas (VSs) represent the most common neoplasms of the cerebellopontine angle in adults. They also constitute roughly 8% of all intracranial tumors. Most VSs (95%) are sporadic and unilateral. In other patients, these tumors are typically associated with neurofibromatosis type II, in which case VSs may also occur bilaterally. Usually, VSs grow in the internal auditory canal (IAC) and cerebellopontine angle. The IAC is home to the superior and inferior vestibular nerve, the cochlear nerve, and the facial nerve. Patients with these tumors often lose their hearing as well as the function of the ipsilateral balance organ. These symptoms are probably caused by pressure on the corresponding nerves but may also arise due to other phenomena, as we will discuss further. Thanks to improvements in imaging studies over the past few decades as well as broader access to magnetic resonance imaging (MRI), tumors are identified earlier and therefore at a smaller stage than in the past.

Preservation of the facial and cochlear nerve function as well as rehabilitation of hearing function (in the case of hearing loss) has increasingly moved into focus over the past years. Measurement of the conduction of the cochlear nerve before, during, and most importantly after tumor removal remains one of the most challenging aspects of this treatment option.

## Vestibular schwannomas

Vestibular schwannomas are benign and usually slowly growing tumors of the vestibulocochlear nerve. Patients present with various primary symptoms such as hearing loss (sudden or progressive), balance issues (sudden or progressive), tinnitus, facial numbness, or facial weakness. Very large tumors can even lead to compression of the brain stem. Due to this variety of possible symptoms, it is important to conduct at least one MRI examination for patients experiencing any of the aforementioned symptoms. In particular, asymmetric hearing loss being the most common symptom of these tumors should prompt caregivers to conduct an MRI of the cerebellopontine angle and inner ear with contrast agent and thin slice thickness. An American guideline recommends imaging studies in cases of interaural hearing differences of more than 10 dB in two frequencies or of more than 15 dB in one frequency [11]. While pure-tone audiograms often only show a slight asymmetry, VSs heavily impact speech understanding, which can only be detected by performing speech recognition tests.

One of the frequently mentioned theories about the development of the aforementioned symptoms is pressure in the IAC with concomitant compression of neuronal structures within the IAC. However, studies have shown that a more probable explanation—at least in early-stage tumors—is the secretion of ototoxic extracellular vesicles by the VS, which can damage cochlear and neural structures alike [27]. A cadaver study examining temporal bones of untreated patients with VS showed that VS causes a significant loss of inner and outer hair cells as well as cochlear neurons. However, this loss did not correlate with tumor size and distance from the cochlea, hence supporting the hypothesis that instead of compression of the vestibulocochlear nerve, ototoxic secretions of VS are responsible for hearing loss [22]. These findings most likely explain why the size, location, and growth rate of a schwannoma do not correlate with the clinically observed degree of hearing loss [5].

The VSs can be graded (grade I to IV) according to the Koos system [15], depending on their location and extension (Table 1).

**Table 1:** Grading system for vestibular schwannomas according to Koos et al. [15]

Grade	Tumor location
Grade I	Intracanalicular
Grade II	Protrusion into cerebellopontine angle (without contact with the brainstem)
Grade III	Occupying cisterna pontocerebellaris (without displacement of the brainstem)
Grade IV	Large tumor with displacement of the brainstem and cranial nerves

## Treatment options for VS

There are various management options for VS, with the three major groups being (a) wait and scan, (b) radiation therapy, and (c) surgery.

A wait-and-scan approach is possible and meaningful for patients with small tumors experiencing few symptoms or having underlying health conditions that preclude surgery. Over time, hearing and balance function are gradually lost due to continuous tumor growth (on average 1–2 mm per year) or its harmful secretions, as mentioned earlier. Patients must therefore be informed that by adopting a wait-and-scan strategy, hearing preservation will most likely not be possible in the long term.

A study published in 2012, in which 212 untreated VS were observed, concluded that 66% of tumors grow over time and about 30% are fast-growing (i.e., doubling in size per year; [26]). Furthermore, while patients with excellent hearing at the time of diagnosis (with a speech discrimination score of 100%) have a higher chance of maintaining useful hearing over time, the majority of patients with only little speech discrimination loss at diagnosis experience continuous deterioration of hearing with only one third of these patients maintaining serviceable hearing over time [28]. Finally, it must be noted that there is only a weak correlation between symptom progression and tumor growth [20]; therefore, many tumors will need treatment at some point. Tumors diagnosed at a certain size cannot be managed with a wait-and-scan approach since further growth can cause serious symptoms and would make treatment even more difficult.

Radiation therapy is mainly conducted using stereotactic radiosurgery (such as a gamma knife). This therapy is possible for patients with T1 or T2 tumors according to the Koos grading scale (Table 1; [15]). In large tumors (T4), radiotherapy is contraindicated due to the associated swelling of the irradiated tissue and consequent risk of pressure on the brainstem. While tumor control rates after gamma knife radiosurgery are as high as 92% after 5 years of follow-up, hearing preservation rates are significantly decreased with only 55% of patients possessing functional hearing 2 years after treatment and 34% 10 years after treatment [9].

**Fig. 1:**
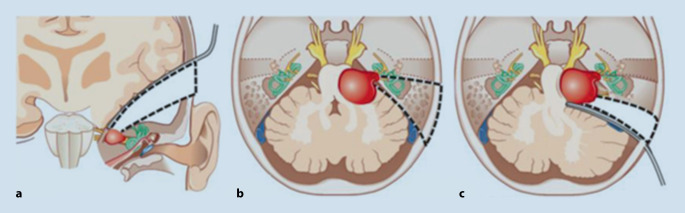
Surgical approaches for vestibular schwannomas extirpation: **a** middle fossa, **b** translabyrinthine, and **c** retrosigmoid approach. (Figure from [1]. Reprinted with permission, © Georg Thieme Group. All rights reserved)

As an alternative option, microsurgical removal of VSs is often performed. Three main surgical approaches are used to resect VSs: subtemporal (middle fossa), translabyrinthine, and retrosigmoid (Fig. 1). The first and last are hearing preservation approaches, which are mainly chosen for patients with serviceable hearing. Tumors that only extend within the IAC have better hearing preservation results than tumors extending into the cerebellopontine angle [25]. In very small VSs, the postoperative hearing preservation rate is as high as 70–85%, according to the literature [23]. A fairly recent meta-analysis reported a 35–49% rate of hearing preservation among patients undergoing retrosigmoid or middle fossa microsurgical resection disregarding tumor size [2].

The three management options are wait and scan, radiation therapy, and surgery

On the contrary, translabyrinthine VS resection has two main advantages for patients: (1) very little pressure on the brain during surgery, as brain retraction can be avoided; (2) possibility to offer patients a simultaneous cochlear implantation (CI) and therefore hearing rehabilitation if cochlear nerve integrity is preserved. Patients with good hearing, however, need to be made aware of the fact that all-natural hearing is certainly lost during this surgery due to the removal of the vestibular part of the inner ear (as seen in Fig. 1).

## Intraoperative assessment of cochlear nerve integrity

A cochlear implant can only be useful to patients when the functional integrity of the cochlear nerve could be preserved during surgery. Cochlear implants can replace the function of damaged hair cells in the inner ear by directly stimulating spiral ganglion neurons; they can, however, not overcome a defective cochlear nerve. Until recently, surgeons assessed the integrity of the nerve visually to decide whether CI could be carried out [4]. Today, objective measurement tools such as electrically evoked auditory brainstem response audiometry (eABR) can and should be used to objectify the surgeon’s opinion and assessment. Facial nerve monitoring is already a well-established method conducted closely during VS resection and can warn surgeons of damage to the facial nerve promptly. Ideally, similar measurement and monitoring techniques would be able to warn surgeons of damage to the cochlear nerve during tumor resection.

eABR enables objective assessment of cochlear nerve responses

Nevertheless, eABR enables objective assessment of cochlear nerve responses at singular timepoints, most importantly before and after tumor removal. The availability of such a useful monitoring technique to intraoperatively assess cochlear nerve integrity should certainly prompt a widespread use of this procedure. To date, however, this technique is only scarcely applied with striking differences concerning the test procedure between different departments. Therefore, we heavily advocate the use of eABR and hope this article and the following explanation of this technique can contribute to its clinical implementation.

## Electrically evoked auditory brainstem response audiometry

Electrically evoked auditory brainstem response is a form of audiometry that assesses the auditory pathway from cochlear to central nervous structures. This sets the eABR apart from various electrocochleography measurements, which are also frequently used for CI patients but only assess the cochlear and peripheral auditory nerve function. In contrast to regular ABR measurements, in eABR, electrical stimuli are presented in proximity to or even within the cochlea. Consequently, neural responses of the nervous auditory pathway can be measured.

**Fig. 2:**
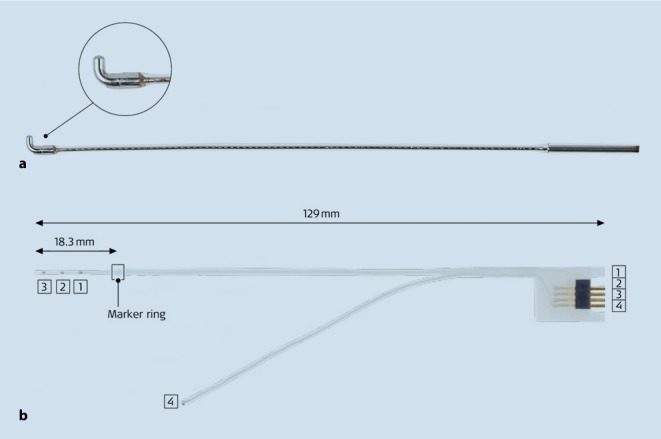
Depiction of the “hockey stick” electrode used for round-window electrically evoked auditory brainstem response (eABR; **a**) and the intracochlear test electrode for intraoperative eABR prior to cochlear implantation (**b**). (Reprinted with permission, © MED-EL, Innsbruck, Austria. All rights reserved)

Electrical stimuli can be delivered via different approaches. A rounded bent-tip (“hockey stick”) electrode (Fig. 2a) can temporarily be placed through the tympanic membrane into the round-window niche and be used to stimulate the auditory nerve [21]. At first, this might strongly resemble the “promontory test,” which was used previously to screen for CI candidacy. However, promontory stimulation is purely based on subjective hearing sensations experienced by the patient in response to the electrical stimulation and was found to possess only a weak prognostic value considering postoperative speech performance. The use of this method was later limited as a screening tool for CI in cases with cochlear abnormalities or retro-labyrinthine deafness [16]. By contrast, eABR delivers objective results and has proven to be far more helpful for determining the excitability of the nervous auditory pathway—especially in patients with inconclusive preoperative audiological tests [8]. Notably, eABR with the “hockey stick” electrode can be performed on patients under local anesthesia, mainly as a basic prognostic marker before VS surgery.

Beside the aforementioned round-window eABR, there is also the possibility to stimulate the auditory nerve from inside the cochlea. A cochlear implant can be used to provide appropriate stimuli for eABR measurements, for example. As in the case of CI after VS extirpation, where cochlear nerve integrity should be confirmed before implantation, a purpose-built intracochlear test electrode (ITE; Fig. 2b) can be used for intracochlear eABR measurements as well [6]. Unlike the cochlear implant, the ITE only has three contacts at an array length of 18 mm and a separate ground electrode. This device is subsequently removed and discarded after measurement.

Disregarding the technique of electrical stimulation, elicited neural responses are measured using electrodes placed on the patient’s head. Contrary to this far-field recording, there is also the possibility of near-field recording of evoked nerve potentials using an electrode placed directly on the auditory nerve (cochlear nerve action potential, CNAP) or via a surface electrode located on the cochlear nucleus (dorsal cochlear nucleus potential, DNAP; [12, 29]; Table 2).

**Table 2:** Overview of different techniques used to assess cochlear nerve integrity

Method	Stimulus	Response	Limitations
Visual inspection	None	Anatomical integrity of the cochlear nerve	Subjective, no physiological assessment, may underestimate lesions of the cochlear nerve
Promontory test	Electrical stimulus via electrode placed onto the promontory	Subjective hearing sensation experienced by the patient	Weak prognostic value; patient must be awake
ABR	Auditory stimulus	Neural responses corresponding to the central auditory pathway	Requires multiple averages resulting in longer acquisition times; cannot be performed in translabyrinthine surgery due to acoustic stimulation
eCAP, also known as (auditory) nerve response telemetry (ART/NRT)	Electrical stimulus with an electrode array inside the cochlea	Neural responses corresponding to the cochlear action potential (CAP) of the first spiral ganglion neuron	Requires an electrode inside the cochlea for stimulation and acquisition; can only assess the function of the first neuron and therefore lesions to the central part of the cochlear nerve may be missed
PS-eABR	Electrical stimulus with a “hockey stick” electrode placed onto the promontory	Neural responses corresponding to the central auditory pathway	Susceptible to artifacts (especially in awake patients), unclear predictive value
Intracochlear eABR	Electrical stimulus with an electrode array inside the cochlea	Neural responses corresponding to the central auditory pathway	Requires surgical access to the inner ear and electrode insertion into the cochlea, far-field measurement
CNAP/DNAP	Acoustic or electrical stimulus	Neural responses of the cochlear nerve action potential (CNAP) or dorsal cochlear nucleus potential (DNAP)	Limited data available, requires invasive placement of surface electrodes onto cochlear nerve or brainstem, acoustic stimulation cannot be used in the case of translabyrinthine surgery

*eABR* electrically evoked auditory brainstem response

Typically, an eABR response consists of seven waves (labeled as I–VII), with each one corresponding to different neural structures along the auditory pathway:

- Wave I: peripheral part of the cochlear nerve
- Wave II: central part of the vestibulocochlear nerve
- Wave III: cochlear nucleus
- Wave IV: olivary body and lateral lemniscus
- Wave V: lateral lemniscus and inferior colliculus
- Waves VI and VII: inferior colliculus and medial geniculate nucleus

Usually, only waves I–V are interpreted clinically. Waves I and II are known to be unstable and prone to artifacts caused by intracochlear stimulation. Additionally, in some cases, only wave V can be reliably detected; therefore this wave is usually the one assessed in the clinical setting. Besides the simple presence of an eABR wave, various other wave characteristics can be interpreted using eABR, such as amplitude, absolute wave latency, or interwave latencies [7].

## eABR in VS surgery

Determining eABR responses has been used to screen for CI candidacy in children (with cochlear malformations; [19]) and can aid when placing cochlear or auditory brainstem implants intraoperatively. After implantation, eABR can be helpful for estimating cochlear nerve function in patients with cochlear implants or to improve fitting parameters (e.g., thresholds or rate of stimulation) for patients with either cochlear or brainstem implants. More recently, as CI is increasingly performed for hearing rehabilitation after VS surgery [30], the application of eABR has expanded to intraoperatively help determine the success of CI in patients with VS [10, 18].

eABR is a reliable tool for assessing cochlear nerve function during VS resection

**Fig. 3:**
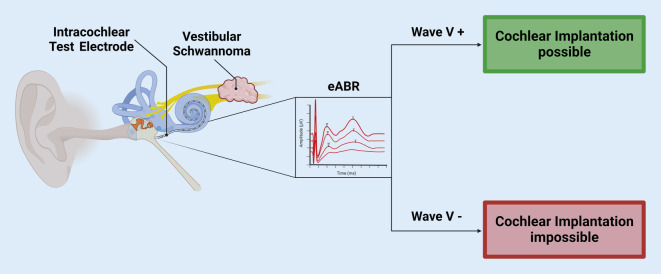
Schematic depiction of the intraoperative decision-making process using electrically evoked auditory brainstem response (*eABR*) as a tool to test cochlear nerve function. (Figure created with biorender.com)

**Fig. 4:**
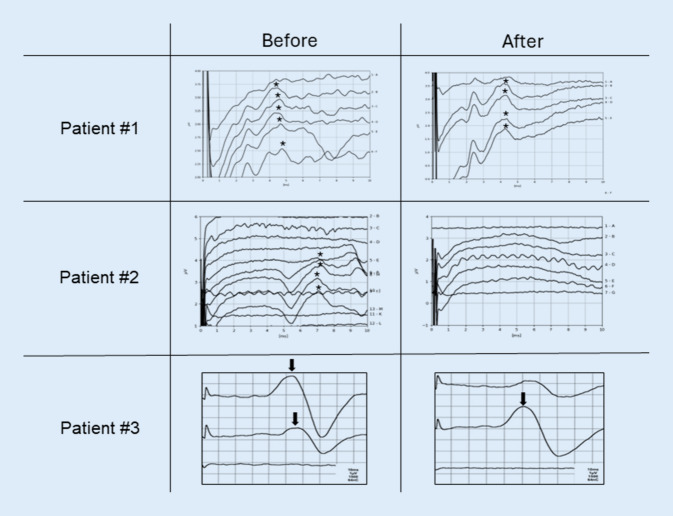
Representative electrically evoked auditory brainstem response (eABR) measurements of different patients before and after vestibular schwannoma removal. *Asterisks* highlight a positive, e.g., clearly reproducible wave V. In the first patient with a positive eABR signal after tumor removal, cochlear implantation was subsequently performed. In the third patient, *arrows* mark a facial nerve artifact

As of today, there is substantial evidence that eABR is a reliable tool for the assessment of cochlear nerve function during VS resection [3]. In this case, eABR measurements before CI are usually performed using an ITE, as described earlier (Fig. 3). Of all the various eABR characteristics to be interpreted, in the case of VS resection, simply the presence of wave V has proven to be a reliable indicator off intact cochlear nerve function [17]. Additionally, a loss of wave V amplitude of more than 50% or an increase in wave V latency of more than 1 ms may act as warning signs for surgeons [14], although no predictive value of these factors for postoperative hearing outcome has been described [13]. Therefore, the presence of a reproducible, clearly detectable eABR wave V after tumor removal (Fig. 4) should prompt surgeons to consider CI. By contrast, there are no general recommendations on what actions must be taken in cases where there is a partial or complete loss of eABR signal. Moreover, systemic and local factors may influence the eABR signal during surgery and mimic cochlear nerve damage. As such, systemic hypotension as well as systemic and local hypothermia may contribute to a loss of eABR amplitude and may increase eABR wave latencies. Therefore, in cases where such alterations of the eABR waves are observed, systemic infusion of or local irrigation with warm fluids to amend hypotension or hypothermia, respectively, may help correct shifts in eABR signals and to distinguish actual cochlear nerve damage [14]. When cochlear nerve damage can be assumed due to a complete loss of an eABR wave V not amendable by the aforementioned procedures, CI may not be successful. Nevertheless, there are reports of patients with negative eABR responses undergoing CI who later achieve auditory perception [10, 18]. However, a few patients with a preserved eABR wave V may not achieve auditory perception after CI. Therefore, it is critical to preoperatively provide appropriate counseling and manage patients’ expectations. Furthermore, due to the current state of knowledge, the final decision on how to manage individual cases must be made by the attending surgeon.

Altogether, performing intraoperative eABR measurements is proven to yield a high accuracy for effectively predicting auditory perception after CI in patients with VS [18]. Nevertheless, as described earlier, CI may still be possible despite the absence of a wave V. Therefore, to further aid decision-making for CI in VS surgery, we have established a scoring system [3]. The scoring system includes preoperative parameters such as tumor size, extension toward the modiolus, residual hearing, and promontory stimulation eABR [3]. Use of eABR with the intracochlear test electrode has been shown to be suitable for the selection of optimal candidates for CI simultaneous to VS extirpation, significantly improving postoperative hearing outcomes and resulting in high implant usage rates [24]. Performing this simultaneous procedure with CI directly after tumor removal provides several benefits over a two-staged approach. These mainly include avoiding partial/complete cochlear ossification impeding future CI and minimizing general perioperative risks associated with a second surgery (e.g., anesthesia-associated complication) or avoiding risks more commonly observed in revision surgeries (e.g., cerebrospinal fluid leakage or cranial nerve deficits).

## Practical conclusion

- Hearing loss is a serious and highly prevalent symptom of vestibular schwannoma (VS). Therefore, hearing preservation and rehabilitation are important therapeutic goals in VS management.
- Cochlear implantation (CI) has emerged as a suitable treatment option for VS-associated hearing loss. Nevertheless, cochlear nerve function must be preserved to ensure proper CI efficacy.
- There are various methods available to assess cochlear nerve integrity before, during, and after VS surgery. Hence, appropriate screening measurements must be selected according to the treatment chosen (e.g., intraoperative electrically evoked auditory brainstem response [eABR] to screen for CI candidacy).
- Intraoperative eABR measurements with an intracochlear electrode have been shown to be a suitable tool for monitoring cochlear nerve status during VS surgery. Furthermore, these measurements facilitate clinical decision-making for CI candidacy.

## Declarations

This article does not contain any studies with human participants or animals performed by any of the authors.
